# Synthesis and crystal structure of La_21_Cr_8−2*a*_Al_*b*_Ge_7−*b*_C_12_ [*a* = 0.22 (2) and *b* = 0.758 (19)]

**DOI:** 10.1107/S2056989016015668

**Published:** 2016-10-11

**Authors:** Jaskarun Pabla, Yuri Janssen, Jack W. Simonson

**Affiliations:** a Department of Physics, Farmingdale State College, Farmingdale, NY 11735, USA

**Keywords:** crystal structure, geometric frustration, multinary chromium carbides

## Abstract

Single crystals of a new multinary chromium carbide, La_21_Cr_8−2*a*_Al_*b*_Ge_7−*b*_C_12_ (henicosa­lanthanum octa­chromium aluminium hexa­germanium dodeca­carbide), were grown from an La-rich self flux and were characterized by single-crystal X-ray diffraction. The face-centered cubic crystal structure is composed of isolated and geometrically frustrated regular Cr tetra­hedra that are co-centered within regular C octa­hedra. These mutually separated Cr_4−a_C_6_ clusters are distributed throughout a three-dimensional framework of Al, Ge, and La. The title compound is isotypic with La_21−δ_Mn_8_ *X*_7_C_12_ and *R*_21_Fe_8_ *X*_7_C_12_ (*R* = La, Ce, Pr; *X* = Al, Bi, Ge, Sn, Sb, Te) and represents the first example of a Cr-based compound with this structure-type.

## Abstract 2

The face-centered cubic crystal structure of a new multinary chromium carbide, La_21_Cr_8−2*a*_Al_*b*_Ge_7−*b*_C_12_, is composed of isolated and geometrically frustrated regular Cr tetra­hedra that are co-centered within regular C octa­hedra. These mutually separated Cr_4−*a*_C_6_ clusters are distributed throughout a three-dimensional framework of Al, Ge, and La.

## Chemical context   

Geometric frustration arises when crystallographic degeneracies lead to the near equalization of competing inter­atomic inter­actions. Often, such frustration results in the suppression to an arbitrarily low temperature of any eventual phase transition to an ordered ground state (Gilbert *et al.*, 2016 ▸). In the simplest case, this phenomenon occurs when three anti­ferromagnetic exchange-coupled Ising spins are arranged on the vertices of an equilateral triangle, their counterbalanced inter­actions thereby precluding the transition to mutually energetically favorable magnetic order. The ability to tune the onset of order *via* geometric frustration has been shown to lead to a variety of intriguing properties, including magnetic monopoles (Pan *et al.*, 2016 ▸), spin ice states (Hirschberger *et al.*, 2015 ▸; Huang *et al.*, 2016 ▸), tricritical phenomena (McNally *et al.*, 2015 ▸), and quantum criticality (Miiller *et al.*, 2016 ▸), with applications ranging from neural networks (Grass *et al.*, 2016 ▸), to quantum computing (Katzgraber *et al.*, 2015 ▸), to unconventional superconductivity (Glasbrenner *et al.*, 2015 ▸). Over the last decades, a class of materials known as pyrochlores has provided a rich ground for studying magnetic frustration due to geometric degeneracies arising from their vertex-linked, regular tetra­hedral building blocks (Gardner *et al.*, 2010 ▸). The structure of the La_21_Fe_8_Sn_7_C_12_ system also consists regular tetra­hedra of Fe, but in this case they are mutually isolated from one another. Here too, geometric frustration has been observed to manifest itself in a spin glass ground state, as inferred from a frequency *f*-dependent cusp in the real part of measurements of ac magnetic susceptibility *χ*′ near temperature *T* = 5 K (Benbow *et al.*, 2009 ▸). On the other hand, if Fe is replaced with Mn as in isostructural La_21_Mn_8_Ge_6.2_Al_0.8_C_12_, similar cusps occurring at *T* = 3 K and 6 K in *χ*′ exhibit no such dependence, even over four orders of magnitude in *f*, suggesting that only local anti­ferromagnetic ordering within the Mn_4_C_6_ cluster arises while the spin glass state remains absent down to *T* = 1.8 K (Zaikina *et al.*, 2011 ▸). With the aim of unveiling a new avenue to explore frustrated states within this class of compounds, we present here the synthesis and crystal structure of a new Cr-based analog that is isostructural and likewise geometrically frustrated, La_21_Cr_8−2*a*_Al_b_Ge_7−*b*_C_12_, [*a* = 0.22 (2), *b* = 0.758 (19)].

## Structural commentary   

**Figure 1:**
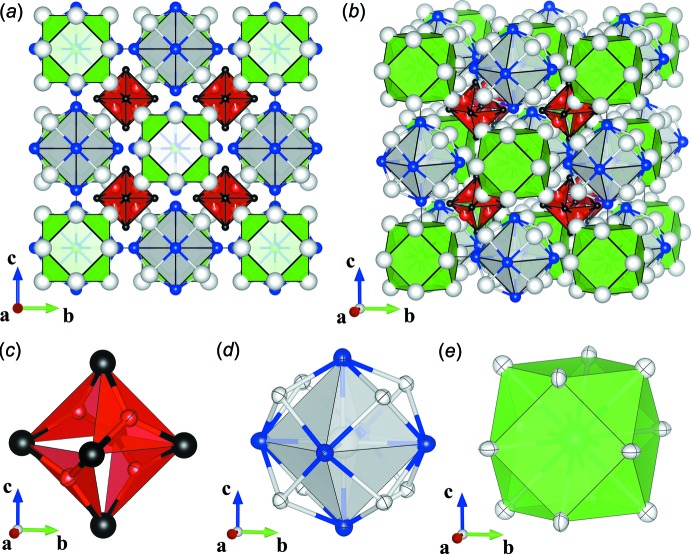
(*a*) A view of the crystal structure of La_21_Cr_8−2*a*_Al_b_Ge_7−**b**_C_12_ along [100]. (*b*) The same crystal structure from an arbitrary view above the *ab* plane. (*c*) Cr-deficient Cr_4−*a*_C_6_ substructure depicted as four tetra­hedrally arranged and vertex-linked CrC_3_ plaquettes. (*d*) La3 coordination polyhedron. (*e*) Al2/Ge2 coordination polyhedron. In all sub-figures, colors are as follows: La (white), Cr (red), Al (green), Ge (blue), and C (black). Polyhedra are colored according to the central element. In *c*–*e*, the ellipsoids correspond to 99% probability.

Fig. 1 ▸ shows a polyhedral representation of the crystal structure of the title compound, the geometrically frustrated substructure of which consists of a Cr-capped regular tetra­hedron enclosed within a C-capped regular octa­hedron. Fig. 1 ▸ *a* is a depiction of the unit cell from along the crystallographic *a* axis, and Fig. 1 ▸ *b* shows the same from a generic angle above the *ab* plane. The structure can be thought to be composed of three building blocks – a geometrically frustrated and Cr-deficient Cr_4−*a*_C_6_ unit (Fig. 1 ▸ *c*), an La_9_Ge_6_ unit (Fig. 1 ▸ *d*), and an La_12_Al_*b*_Ge_1−*b*_ unit (Fig. 1 ▸ *e*). These substructures are arranged on four inter­penetrating face-centered cubic lattices that originate within the unit cell at (¼, ¼, ¼) and (¾, ¼, ¼) for the Cr_4−*a*_C_6_ unit, (½, 0, 0) for the La_9_Ge_6_ unit, and (0, 0, 0) for the La_12_Al_*b*_Ge_1-*b*_ unit. Accordingly, La_21_Cr_8−2*a*_Al_*b*_Ge_7−*b*_C_12_ adopts a structure that is effectively a polyatomic analog of the Heusler structure (Graf *et al.*, 2011 ▸) with composition *X*_2_ *YZ*, where *X* = Cr_4−*a*_C_6_, *Y* = La_9_Ge_6_, and *Z* = La_12_Al_*b*_Ge_1−*b*_ units. Taken together with the appropriate site occupancies, the title composition is thus obtained as *X*_2_ *YZ* = La_21_Cr_8−2a_Al_*b*_Ge_7−*b*_C_12_.

The geometrically frustrated Cr-deficient Cr_4−*a*_C_6_ unit shown in Fig. 1 ▸ *c* is composed of a single inequivalent Cr position and a single C position. Accordingly, nearest neighbor Cr—C distances are uniformly 1.949 (5) Å, in good agreement with nearest neighbor distances in binary Cr carbides. Likewise, all Cr—Cr distances within the substructure are similarly identical at 2.4821 (9) Å, only slightly smaller than the 2.512 Å nearest neighbor distance observed in Cr metal (Gorbunoff *et al.*, 2009 ▸). Perhaps more inter­esting, however, is this relative proximity when compared with the 2.878 Å that separates neighboring Cr in the frustrated Kagomé planes of SrCr_8−*x*_Ga_4+*x*_O_19_, a seminal example of a geometrically frustrated magnetic system (Broholm *et al.*, 1990 ▸).

The remaining substructures, namely the La_9_Ge_6_ unit shown in Fig. 1 ▸ *d* and the La_12_Al_*b*_Ge_1−*b*_ unit shown in Fig. 1 ▸ *e* form cages about their central La3 and Al2/Ge2 sites respectively. The cage-like nature of this configuration is clear from the large anisotropic displacement parameters *U*_eq_ corresponding to these two central sites, as has been previously observed in isostructural materials (Benbow *et al.*, 2009 ▸; Zaikina *et al.*, 2011 ▸). These sites are likely characterized by strong rattling modes of the central loosely bound atom, such as is observed in skutterudite compounds (Sergueev *et al.*, 2015 ▸). Not surprisingly, the distance between central La3 and its nearest neighbor Ge1 is a rather long, 3.41450 (13) Å. The central Al2/Ge2 site is even further – 3.8858 (2) Å from its nearest neighbor La1. A brief review of the crystallographic literature finds nearest neighbor bond lengths in La—Ge binaries to be typically on the order of only 3.0 to 3.2 Å, far smaller than either of these distances, which lends credence to the emerging picture of a stuffed, skutterudite-like arrangement.

## Synthesis and crystallization   

La_21_Cr_8−2*a*_Al_*b*_Ge_7−*b*_C_12_ crystals were grown from a self flux of excess La (Alfa Aesar, 00175) and the following chemicals: Cr (Alfa Aesar, 38494), Ge (Strategic Metal, SM1301-B), and graphite (McMaster-Carr 9121K71) in an La:Cr:Ge:C atomic ratio of 561:214:76:149. The growth process was carried out in Al_2_O_3_ crucibles sealed within fused quartz ampoules under high purity Ar gas. Ampoules were heated to 1423 K over a period of four h, left to soak at that temperature for an additional four h, and cooled to 1173 K over 50 h to induce nucleation and to promote crystal growth. The ampoule was then quickly centrifuged at 2000 r.p.m. for several seconds to separate the solid crystals from the liquid La-rich solution. Crystals took the form of well-faceted tablets with metallic luster.

## Refinement details   

Details regarding the crystal itself, as well as data collection and structural refinement are presented in Table 1 ▸. No evidence for twin domains was observed, and all sites with the exception of C were refined with anisotropic displacement parameters. Here permitting anisotropic displacement parameters did not appreciably improve the refinement. Two reflections, (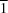 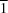 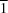) and (00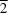), required manual culling due to beamstop clipping.

The refinement was improved when the Ge2 site was permitted to be mixed with Al. In this case, Al and Ge coord­inates and displacement parameters were constrained to be equal, and the sum of the Al and Ge occupancies was constrained to unity. The refined Al:Ge ratio 0.758 (19):6.242 (19) is in excellent agreement with observed ratios of 0.83 (2):6.17 (2) in La_21_MnAl_*b*_Ge_7−*b*_C_12_ (Zaikina *et al.*, 2011 ▸) and somewhat lower than the reported ratio of 2.1:4.9 in La_21_FeAl_*b*_Ge_7-*b*_C_12_ (Benbow *et al.*, 2009 ▸). Like the Mn-based analog, however, we observe no evidence to suggest that the Ge1 site is mixed, as was the case with the more Al-rich La_21_FeAl_*b*_Ge_7−*b*_C_12_. Regardless of any qu­anti­tative differences, the potential for Al – apparently extracted by an La-rich flux from Al_2_O_3_ growth crucibles – to mix with Ge appears to be a universal phenomenon in this class of compounds. It remains unclear if Al is required to stabilize the Ge-containing examples of these phases, which have not been reported in its absence.

In addition to mixing on the Al2/Ge2 site, excess charge was observed in Fourier maps when the Cr site was constrained to full occupancy, and the refinement was substanti­ally improved when this parameter was subsequently freed. Permitting instead partial occupancy of Al on the Cr site did not appreciably improve the refinement. No evidence for mixed or non-unity occupancy was found for any of the La sites, despite previously published density functional theory calculations that found a composition of La_20_Mn_8_Te_7_C_12_ to be stabilized by the shift of the Fermi energy to a pseudogap in the density of states (Zaikina *et al.*, 2011 ▸). Our final refined composition is then La_21_Cr_8−2*a*_Al_*b*_Ge_7−*b*_C_12_ with the occupancy parameters *a* = 0.22 (2) and *b* = 0.758 (19).

## Supplementary Material

Crystal structure: contains datablock(s) global, I. DOI: 10.1107/S2056989016015668/pk2592sup1.cif

Structure factors: contains datablock(s) global, I. DOI: 10.1107/S2056989016015668/pk2592Isup2.hkl

CCDC reference: 1508202

Additional supporting information: crystallographic information; 3D view; checkCIF report

## Figures and Tables

**Table 1:** Experimental details

Crystal data	
Chemical formula	La_21_Cr_7.556_Al_0.758_Ge_6.242_C_12_
*M*_r_	3927.6
Crystal system, space group	Cubic, *F* *m* 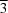 *m*
Temperature (K)	294
*a* (Å)	16.4048 (6)
*V* (Å^3^)	4414.8 (5)
*Z*	4
Radiation type	Mo *K*α
μ (mm^−1^)	25.76
Crystal size (mm)	0.12 × 0.11 × 0.07

Data collection	
Diffractometer	Bruker APEXII CCD
Absorption correction	Numerical (*SADABS*; Bruker, 2008 ▸)
*T*_min_, *T*_max_	0.342, 0.527
No. of measured, independent and observed [*I* > 3σ(*I*)] reflections	40979, 328, 321
*R*_int_	0.041
(sin θ/λ)_max_ (Å^−1^)	0.670

Refinement	
*R*[*F* > 3σ(*F*)], *wR*(*F*), *S*	0.012, 0.045, 1.91
No. of reflections	328
No. of parameters	21
Δρ_max_, Δρ_min_ (e Å^−3^)	1.07, −0.83

Computer programs: *APEX2* and *SAINT* (Bruker, 2007 ▸), *SUPERFLIP* (Palatinus & Chapuis, 2007 ▸), *JANA2006* (Petřìček *et al.*, 2014 ▸), *VESTA* (Momma & Izumi, 2011 ▸) and *publCIF* (Westrip, 2010 ▸).

## supplementary crystallographic information

### Crystal data

**Table:**

La_21_Cr_7.556_Al_0.758_Ge_6.242_C_12_	*D*_x_ = 5.909 Mg m^−^^3^
*M**_r_* = 3927.6	Mo *K*α radiation, λ = 0.71073 Å
Cubic, *F**m*3*m*	Cell parameters from 9327 reflections
Hall symbol: -F 4 2 3	θ = 5.0–56.7°
*a* = 16.4048 (6) Å	µ = 25.76 mm^−^^1^
*V* = 4414.8 (5) Å^3^	*T* = 294 K
*Z* = 4	Plate, metallic_black
*F*(000) = 6639.4	0.12 × 0.11 × 0.07 mm

### Data collection

**Table:**

Bruker APEXII CCD diffractometer	328 independent reflections
Radiation source: X-ray tube	321 reflections with *I* > 3σ(*I*)
Graphite monochromator	*R*_int_ = 0.041
ω and φ scans	θ_max_ = 28.4°, θ_min_ = 2.2°
Absorption correction: numerical (*SADABS*; Bruker, 2008)	*h* = −21→21
*T*_min_ = 0.342, *T*_max_ = 0.527	*k* = −21→21
40979 measured reflections	*l* = −21→21

### Refinement

**Table:**

Refinement on *F*^2^	1 constraint
*R*[*F* > 3σ(*F*)] = 0.012	Weighting scheme based on measured s.u.'s *w* = 1/(σ^2^(*I*) + 0.0004*I*^2^)
*wR*(*F*) = 0.045	(Δ/σ)_max_ = 0.015
*S* = 1.91	Δρ_max_ = 1.07 e Å^−^^3^
328 reflections	Δρ_min_ = −0.83 e Å^−^^3^
21 parameters	Extinction correction: B-C type 2 (Becker & Coppens, 1974)
0 restraints	Extinction coefficient: 810 (150)

### Fractional atomic coordinates and isotropic or equivalent isotropic displacement parameters (Å^2^)

**Table:**

	*x*	*y*	*z*	*U*_iso_*/*U*_eq_	Occ. (<1)
La1	0	0.167496 (14)	0.167496 (14)	0.01125 (10)	
La2	0.369379 (15)	0.369379 (15)	0.369379 (15)	0.00984 (9)	
La3	0.5	0.5	0.5	0.0384 (3)	
Ge1	0.29186 (6)	0	0	0.0121 (2)	
Cr1	0.19651 (4)	0.19651 (4)	0.19651 (4)	0.0068 (2)	0.944 (5)
Ge2	0	0	0	0.0323 (12)	0.242 (19)
Al2	0	0	0	0.0323 (12)	0.758 (19)
C1	0.1049 (4)	0.25	0.25	0.0127 (11)*	

### Atomic displacement parameters (Å^2^)

**Table:**

	*U*^11^	*U*^22^	*U*^33^	*U*^12^	*U*^13^	*U*^23^
La1	0.0074 (2)	0.01315 (17)	0.01315 (17)	0	0	0.00046 (13)
La2	0.00984 (15)	0.00984 (15)	0.00984 (15)	−0.00052 (8)	−0.00052 (8)	−0.00052 (8)
La3	0.0384 (4)	0.0384 (4)	0.0384 (4)	0	0	0
Ge1	0.0139 (5)	0.0112 (3)	0.0112 (3)	0	0	0
Cr1	0.0068 (3)	0.0068 (3)	0.0068 (3)	0.0008 (2)	0.0008 (2)	0.0008 (2)
Ge2	0.032 (2)	0.032 (2)	0.032 (2)	0	0	0
Al2	0.032 (2)	0.032 (2)	0.032 (2)	0	0	0

### Geometric parameters (Å, º)

**Table:**

La1—La1^i^	3.8282 (4)	La2—Ge1^xiii^	3.2864 (5)
La1—La1^ii^	3.8859 (3)	La2—Ge1^xiv^	3.2864 (5)
La1—La1^iii^	3.8859 (3)	La2—Cr1^vi^	3.2216 (7)
La1—La1^iv^	3.8859 (3)	La2—Cr1^vii^	3.2216 (7)
La1—La1^v^	3.8859 (3)	La2—Cr1^i^	3.2216 (7)
La1—La2^vi^	3.9907 (4)	La2—C1^vi^	2.8015 (9)
La1—La2^vii^	3.9907 (4)	La2—C1^xv^	2.8015 (9)
La1—La2^viii^	3.9907 (4)	La2—C1^xvi^	2.8015 (9)
La1—La2^ix^	3.9907 (4)	Cr1—Cr1^vi^	2.4821 (9)
La1—Cr1	3.2932 (7)	Cr1—Cr1^vii^	2.4821 (9)
La1—Cr1^x^	3.2932 (7)	Cr1—Cr1^i^	2.4821 (9)
La1—C1	2.574 (4)	Cr1—C1	1.949 (5)
La1—C1^xi^	2.574 (4)	Cr1—C1^ii^	1.949 (5)
La2—La3	3.7115 (3)	Cr1—C1^iv^	1.949 (5)
La2—Ge1^xii^	3.2864 (5)		
			
La1^i^—La1—La1^ii^	120.000 (6)	La3—La2—Cr1^vii^	153.589 (14)
La1^i^—La1—La1^iii^	120.000 (6)	La3—La2—Cr1^i^	153.589 (14)
La1^i^—La1—La1^iv^	120.000 (6)	La3—La2—C1^vi^	136.07 (12)
La1^i^—La1—La1^v^	120.000 (6)	La3—La2—C1^xv^	136.07 (12)
La1^i^—La1—La2^vi^	61.339 (6)	La3—La2—C1^xvi^	136.07 (12)
La1^i^—La1—La2^vii^	61.339 (6)	Ge1^xii^—La2—Ge1^xiii^	94.557 (15)
La1^i^—La1—La2^viii^	61.339 (6)	Ge1^xii^—La2—Ge1^xiv^	94.557 (15)
La1^i^—La1—La2^ix^	61.339 (6)	Ge1^xii^—La2—Cr1^vi^	131.521 (19)
La1^i^—La1—Cr1	78.207 (12)	Ge1^xii^—La2—Cr1^vii^	131.521 (19)
La1^i^—La1—Cr1^x^	78.207 (12)	Ge1^xii^—La2—Cr1^i^	95.56 (2)
La1^i^—La1—C1	41.96 (10)	Ge1^xii^—La2—C1^vi^	165.90 (12)
La1^i^—La1—C1^xi^	41.96 (10)	Ge1^xii^—La2—C1^xv^	95.00 (8)
La1^ii^—La1—La1^iii^	90.000 (7)	Ge1^xii^—La2—C1^xvi^	95.00 (8)
La1^ii^—La1—La1^iv^	60.000 (5)	Ge1^xiii^—La2—Ge1^xiv^	94.557 (15)
La1^ii^—La1—La1^v^	120.000 (7)	Ge1^xiii^—La2—Cr1^vi^	131.521 (19)
La1^ii^—La1—La2^vi^	60.865 (6)	Ge1^xiii^—La2—Cr1^vii^	95.56 (2)
La1^ii^—La1—La2^vii^	101.955 (5)	Ge1^xiii^—La2—Cr1^i^	131.521 (19)
La1^ii^—La1—La2^viii^	165.127 (7)	Ge1^xiii^—La2—C1^vi^	95.00 (8)
La1^ii^—La1—La2^ix^	105.813 (7)	Ge1^xiii^—La2—C1^xv^	165.90 (12)
La1^ii^—La1—Cr1	53.844 (12)	Ge1^xiii^—La2—C1^xvi^	95.00 (8)
La1^ii^—La1—Cr1^x^	142.596 (13)	Ge1^xiv^—La2—Cr1^vi^	95.56 (2)
La1^ii^—La1—C1	84.21 (8)	Ge1^xiv^—La2—Cr1^vii^	131.521 (19)
La1^ii^—La1—C1^xi^	147.63 (4)	Ge1^xiv^—La2—Cr1^i^	131.521 (19)
La1^iii^—La1—La1^iv^	120.000 (7)	Ge1^xiv^—La2—C1^vi^	95.00 (8)
La1^iii^—La1—La1^v^	60.000 (5)	Ge1^xiv^—La2—C1^xv^	95.00 (8)
La1^iii^—La1—La2^vi^	105.813 (7)	Ge1^xiv^—La2—C1^xvi^	165.90 (12)
La1^iii^—La1—La2^vii^	165.127 (7)	Cr1^vi^—La2—Cr1^vii^	45.314 (17)
La1^iii^—La1—La2^viii^	101.955 (5)	Cr1^vi^—La2—Cr1^i^	45.314 (17)
La1^iii^—La1—La2^ix^	60.865 (6)	Cr1^vi^—La2—C1^vi^	36.93 (9)
La1^iii^—La1—Cr1	142.596 (13)	Cr1^vi^—La2—C1^xv^	36.93 (9)
La1^iii^—La1—Cr1^x^	53.844 (12)	Cr1^vi^—La2—C1^xvi^	70.34 (12)
La1^iii^—La1—C1	147.63 (4)	Cr1^vii^—La2—Cr1^i^	45.314 (17)
La1^iii^—La1—C1^xi^	84.21 (8)	Cr1^vii^—La2—C1^vi^	36.93 (9)
La1^iv^—La1—La1^v^	90.000 (7)	Cr1^vii^—La2—C1^xv^	70.34 (12)
La1^iv^—La1—La2^vi^	101.955 (5)	Cr1^vii^—La2—C1^xvi^	36.93 (9)
La1^iv^—La1—La2^vii^	60.865 (6)	Cr1^i^—La2—C1^vi^	70.34 (12)
La1^iv^—La1—La2^viii^	105.813 (7)	Cr1^i^—La2—C1^xv^	36.93 (9)
La1^iv^—La1—La2^ix^	165.127 (7)	Cr1^i^—La2—C1^xvi^	36.93 (9)
La1^iv^—La1—Cr1	53.844 (12)	C1^vi^—La2—C1^xv^	73.86 (13)
La1^iv^—La1—Cr1^x^	142.596 (13)	C1^vi^—La2—C1^xvi^	73.86 (13)
La1^iv^—La1—C1	84.21 (8)	C1^xv^—La2—C1^xvi^	73.86 (13)
La1^iv^—La1—C1^xi^	147.63 (4)	La2—La3—La2^xix^	109.471 (6)
La1^v^—La1—La2^vi^	165.127 (7)	La2—La3—La2^xx^	109.471 (6)
La1^v^—La1—La2^vii^	105.813 (7)	La2—La3—La2^xxi^	109.471 (6)
La1^v^—La1—La2^viii^	60.865 (6)	La2—La3—La2^xxii^	70.529 (6)
La1^v^—La1—La2^ix^	101.955 (5)	La2—La3—La2^xxiii^	180.0 (5)
La1^v^—La1—Cr1	142.596 (13)	La2—La3—La2^xxiv^	70.529 (6)
La1^v^—La1—Cr1^x^	53.844 (12)	La2—La3—La2^xxv^	70.529 (6)
La1^v^—La1—C1	147.63 (4)	La2^xix^—La3—La2^xx^	109.471 (6)
La1^v^—La1—C1^xi^	84.21 (8)	La2^xix^—La3—La2^xxi^	109.471 (6)
La2^vi^—La1—La2^vii^	87.896 (6)	La2^xix^—La3—La2^xxii^	180.0 (5)
La2^vi^—La1—La2^viii^	122.677 (8)	La2^xix^—La3—La2^xxiii^	70.529 (6)
La2^vi^—La1—La2^ix^	64.952 (7)	La2^xix^—La3—La2^xxiv^	70.529 (6)
La2^vi^—La1—Cr1	51.418 (12)	La2^xix^—La3—La2^xxv^	70.529 (6)
La2^vi^—La1—Cr1^x^	115.315 (13)	La2^xx^—La3—La2^xxi^	109.471 (6)
La2^vi^—La1—C1	44.302 (12)	La2^xx^—La3—La2^xxii^	70.529 (6)
La2^vi^—La1—C1^xi^	90.13 (7)	La2^xx^—La3—La2^xxiii^	70.529 (6)
La2^vii^—La1—La2^viii^	64.952 (7)	La2^xx^—La3—La2^xxiv^	180.0 (5)
La2^vii^—La1—La2^ix^	122.677 (8)	La2^xx^—La3—La2^xxv^	70.529 (6)
La2^vii^—La1—Cr1	51.418 (12)	La2^xxi^—La3—La2^xxii^	70.529 (6)
La2^vii^—La1—Cr1^x^	115.315 (13)	La2^xxi^—La3—La2^xxiii^	70.529 (6)
La2^vii^—La1—C1	44.302 (12)	La2^xxi^—La3—La2^xxiv^	70.529 (6)
La2^vii^—La1—C1^xi^	90.13 (7)	La2^xxi^—La3—La2^xxv^	180.0 (5)
La2^viii^—La1—La2^ix^	87.896 (6)	La2^xxii^—La3—La2^xxiii^	109.471 (6)
La2^viii^—La1—Cr1	115.314 (13)	La2^xxii^—La3—La2^xxiv^	109.471 (6)
La2^viii^—La1—Cr1^x^	51.418 (12)	La2^xxii^—La3—La2^xxv^	109.471 (6)
La2^viii^—La1—C1	90.13 (7)	La2^xxiii^—La3—La2^xxiv^	109.471 (6)
La2^viii^—La1—C1^xi^	44.302 (12)	La2^xxiii^—La3—La2^xxv^	109.471 (6)
La2^ix^—La1—Cr1	115.314 (13)	La2^xxiv^—La3—La2^xxv^	109.471 (6)
La2^ix^—La1—Cr1^x^	51.418 (12)	La2^xxvi^—Ge1—La2^i^	134.47 (3)
La2^ix^—La1—C1	90.13 (7)	La2^xxvi^—Ge1—La2^xxvii^	81.389 (12)
La2^ix^—La1—C1^xi^	44.302 (12)	La2^xxvi^—Ge1—La2^xxviii^	81.389 (12)
Cr1—La1—Cr1^x^	156.414 (18)	La2^i^—Ge1—La2^xxvii^	81.389 (12)
Cr1—La1—C1	36.25 (10)	La2^i^—Ge1—La2^xxviii^	81.389 (12)
Cr1—La1—C1^xi^	120.16 (10)	La2^xxvii^—Ge1—La2^xxviii^	134.47 (3)
Cr1^x^—La1—C1	120.16 (10)	La1—Cr1—La1^ii^	72.313 (15)
Cr1^x^—La1—C1^xi^	36.25 (10)	La1—Cr1—La1^iv^	72.313 (15)
C1—La1—C1^xi^	83.91 (14)	La1—Cr1—La2^vi^	75.541 (15)
La1^vi^—La2—La1^vii^	57.323 (6)	La1—Cr1—La2^vii^	75.541 (15)
La1^vi^—La2—La1^xv^	113.653 (7)	La1—Cr1—La2^i^	139.88 (2)
La1^vi^—La2—La1^xvii^	58.269 (6)	La1—Cr1—Cr1^vi^	142.60 (3)
La1^vi^—La2—La1^xvi^	113.653 (7)	La1—Cr1—Cr1^vii^	142.60 (3)
La1^vi^—La2—La1^xviii^	150.254 (8)	La1—Cr1—Cr1^i^	101.79 (3)
La1^vi^—La2—La3	104.871 (7)	La1—Cr1—C1	51.34 (11)
La1^vi^—La2—Ge1^xii^	147.964 (10)	La1—Cr1—C1^ii^	113.23 (9)
La1^vi^—La2—Ge1^xiii^	97.605 (8)	La1—Cr1—C1^iv^	113.23 (9)
La1^vi^—La2—Ge1^xiv^	55.083 (13)	La1^ii^—Cr1—La1^iv^	72.313 (15)
La1^vi^—La2—Cr1^vi^	53.041 (13)	La1^ii^—Cr1—La2^vi^	75.541 (15)
La1^vi^—La2—Cr1^vii^	76.602 (13)	La1^ii^—Cr1—La2^vii^	139.88 (2)
La1^vi^—La2—Cr1^i^	98.244 (13)	La1^ii^—Cr1—La2^i^	75.541 (15)
La1^vi^—La2—C1^vi^	39.92 (8)	La1^ii^—Cr1—Cr1^vi^	142.60 (3)
La1^vi^—La2—C1^xv^	79.52 (2)	La1^ii^—Cr1—Cr1^vii^	101.79 (3)
La1^vi^—La2—C1^xvi^	113.23 (10)	La1^ii^—Cr1—Cr1^i^	142.60 (3)
La1^vii^—La2—La1^xv^	150.254 (8)	La1^ii^—Cr1—C1	113.23 (9)
La1^vii^—La2—La1^xvii^	113.653 (7)	La1^ii^—Cr1—C1^ii^	51.34 (11)
La1^vii^—La2—La1^xvi^	58.269 (6)	La1^ii^—Cr1—C1^iv^	113.23 (9)
La1^vii^—La2—La1^xviii^	113.653 (7)	La1^iv^—Cr1—La2^vi^	139.88 (2)
La1^vii^—La2—La3	104.871 (7)	La1^iv^—Cr1—La2^vii^	75.541 (15)
La1^vii^—La2—Ge1^xii^	147.964 (10)	La1^iv^—Cr1—La2^i^	75.541 (15)
La1^vii^—La2—Ge1^xiii^	55.083 (13)	La1^iv^—Cr1—Cr1^vi^	101.79 (3)
La1^vii^—La2—Ge1^xiv^	97.605 (8)	La1^iv^—Cr1—Cr1^vii^	142.60 (3)
La1^vii^—La2—Cr1^vi^	76.602 (13)	La1^iv^—Cr1—Cr1^i^	142.60 (3)
La1^vii^—La2—Cr1^vii^	53.041 (13)	La1^iv^—Cr1—C1	113.23 (9)
La1^vii^—La2—Cr1^i^	98.244 (13)	La1^iv^—Cr1—C1^ii^	113.23 (9)
La1^vii^—La2—C1^vi^	39.92 (8)	La1^iv^—Cr1—C1^iv^	51.34 (11)
La1^vii^—La2—C1^xv^	113.23 (10)	La2^vi^—Cr1—La2^vii^	118.56 (2)
La1^vii^—La2—C1^xvi^	79.52 (2)	La2^vi^—Cr1—La2^i^	118.56 (2)
La1^xv^—La2—La1^xvii^	57.323 (6)	La2^vi^—Cr1—Cr1^vi^	118.32 (3)
La1^xv^—La2—La1^xvi^	113.653 (7)	La2^vi^—Cr1—Cr1^vii^	67.34 (2)
La1^xv^—La2—La1^xviii^	58.269 (6)	La2^vi^—Cr1—Cr1^i^	67.34 (2)
La1^xv^—La2—La3	104.871 (7)	La2^vi^—Cr1—C1	59.74 (2)
La1^xv^—La2—Ge1^xii^	55.083 (13)	La2^vi^—Cr1—C1^ii^	59.74 (2)
La1^xv^—La2—Ge1^xiii^	147.964 (10)	La2^vi^—Cr1—C1^iv^	168.77 (11)
La1^xv^—La2—Ge1^xiv^	97.605 (8)	La2^vii^—Cr1—La2^i^	118.56 (2)
La1^xv^—La2—Cr1^vi^	76.602 (13)	La2^vii^—Cr1—Cr1^vi^	67.34 (2)
La1^xv^—La2—Cr1^vii^	98.244 (13)	La2^vii^—Cr1—Cr1^vii^	118.32 (3)
La1^xv^—La2—Cr1^i^	53.041 (13)	La2^vii^—Cr1—Cr1^i^	67.34 (2)
La1^xv^—La2—C1^vi^	113.23 (10)	La2^vii^—Cr1—C1	59.74 (2)
La1^xv^—La2—C1^xv^	39.92 (8)	La2^vii^—Cr1—C1^ii^	168.77 (11)
La1^xv^—La2—C1^xvi^	79.52 (2)	La2^vii^—Cr1—C1^iv^	59.74 (2)
La1^xvii^—La2—La1^xvi^	150.254 (8)	La2^i^—Cr1—Cr1^vi^	67.34 (2)
La1^xvii^—La2—La1^xviii^	113.653 (7)	La2^i^—Cr1—Cr1^vii^	67.34 (2)
La1^xvii^—La2—La3	104.871 (7)	La2^i^—Cr1—Cr1^i^	118.32 (3)
La1^xvii^—La2—Ge1^xii^	97.605 (8)	La2^i^—Cr1—C1	168.77 (11)
La1^xvii^—La2—Ge1^xiii^	147.964 (10)	La2^i^—Cr1—C1^ii^	59.74 (2)
La1^xvii^—La2—Ge1^xiv^	55.083 (13)	La2^i^—Cr1—C1^iv^	59.74 (2)
La1^xvii^—La2—Cr1^vi^	53.041 (13)	Cr1^vi^—Cr1—Cr1^vii^	60.00 (3)
La1^xvii^—La2—Cr1^vii^	98.244 (13)	Cr1^vi^—Cr1—Cr1^i^	60.00 (3)
La1^xvii^—La2—Cr1^i^	76.602 (13)	Cr1^vi^—Cr1—C1	103.11 (10)
La1^xvii^—La2—C1^vi^	79.52 (2)	Cr1^vi^—Cr1—C1^ii^	103.11 (10)
La1^xvii^—La2—C1^xv^	39.92 (8)	Cr1^vi^—Cr1—C1^iv^	50.45 (11)
La1^xvii^—La2—C1^xvi^	113.23 (10)	Cr1^vii^—Cr1—Cr1^i^	60.00 (3)
La1^xvi^—La2—La1^xviii^	57.323 (6)	Cr1^vii^—Cr1—C1	103.11 (10)
La1^xvi^—La2—La3	104.871 (7)	Cr1^vii^—Cr1—C1^ii^	50.45 (11)
La1^xvi^—La2—Ge1^xii^	97.605 (8)	Cr1^vii^—Cr1—C1^iv^	103.11 (10)
La1^xvi^—La2—Ge1^xiii^	55.083 (13)	Cr1^i^—Cr1—C1	50.45 (11)
La1^xvi^—La2—Ge1^xiv^	147.964 (10)	Cr1^i^—Cr1—C1^ii^	103.11 (10)
La1^xvi^—La2—Cr1^vi^	98.244 (13)	Cr1^i^—Cr1—C1^iv^	103.11 (10)
La1^xvi^—La2—Cr1^vii^	53.041 (13)	C1—Cr1—C1^ii^	119.45 (4)
La1^xvi^—La2—Cr1^i^	76.602 (13)	C1—Cr1—C1^iv^	119.45 (4)
La1^xvi^—La2—C1^vi^	79.52 (2)	C1^ii^—Cr1—C1^iv^	119.45 (4)
La1^xvi^—La2—C1^xv^	113.23 (10)	La1—C1—La1^i^	96.09 (19)
La1^xvi^—La2—C1^xvi^	39.92 (8)	La1—C1—La2^vi^	95.78 (7)
La1^xviii^—La2—La3	104.871 (7)	La1—C1—La2^vii^	95.78 (7)
La1^xviii^—La2—Ge1^xii^	55.083 (13)	La1—C1—Cr1	92.41 (2)
La1^xviii^—La2—Ge1^xiii^	97.605 (8)	La1—C1—Cr1^i^	171.5 (2)
La1^xviii^—La2—Ge1^xiv^	147.964 (10)	La1^i^—C1—La2^vi^	95.78 (7)
La1^xviii^—La2—Cr1^vi^	98.244 (13)	La1^i^—C1—La2^vii^	95.78 (7)
La1^xviii^—La2—Cr1^vii^	76.602 (13)	La1^i^—C1—Cr1	171.5 (2)
La1^xviii^—La2—Cr1^i^	53.041 (13)	La1^i^—C1—Cr1^i^	92.41 (2)
La1^xviii^—La2—C1^vi^	113.23 (10)	La2^vi^—C1—La2^vii^	162.7 (2)
La1^xviii^—La2—C1^xv^	79.52 (2)	La2^vi^—C1—Cr1	83.33 (10)
La1^xviii^—La2—C1^xvi^	39.92 (8)	La2^vi^—C1—Cr1^i^	83.33 (10)
La3—La2—Ge1^xii^	58.029 (15)	La2^vii^—C1—Cr1	83.33 (10)
La3—La2—Ge1^xiii^	58.029 (15)	La2^vii^—C1—Cr1^i^	83.33 (10)
La3—La2—Ge1^xiv^	58.029 (15)	Cr1—C1—Cr1^i^	79.1 (2)
La3—La2—Cr1^vi^	153.589 (14)		

Symmetry codes: (i) *x*, −*y*+1/2, −*z*+1/2; (ii) *z*, *x*, *y*; (iii) −*z*, −*x*, *y*; (iv) *y*, *z*, *x*; (v) −*y*, *z*, −*x*; (vi) −*x*+1/2, −*y*+1/2, *z*; (vii) −*x*+1/2, *y*, −*z*+1/2; (viii) *y*−1/2, *x*, −*z*+1/2; (ix) *y*−1/2, −*x*+1/2, *z*; (x) −*y*, *x*, *z*; (xi) −*x*, *z*, *y*; (xii) *x*, *y*+1/2, *z*+1/2; (xiii) *z*+1/2, *x*, *y*+1/2; (xiv) *y*+1/2, *z*+1/2, *x*; (xv) *z*, −*x*+1/2, −*y*+1/2; (xvi) −*y*+1/2, *z*, −*x*+1/2; (xvii) −*z*+1/2, −*x*+1/2, *y*; (xviii) *y*, −*z*+1/2, −*x*+1/2; (xix) −*x*+1, −*y*+1, *z*; (xx) −*x*+1, *y*, −*z*+1; (xxi) *x*, −*y*+1, −*z*+1; (xxii) *y*, *x*, −*z*+1; (xxiii) −*y*+1, −*x*+1, −*z*+1; (xxiv) *y*, −*x*+1, *z*; (xxv) −*y*+1, *x*, *z*; (xxvi) *x*, *y*−1/2, *z*−1/2; (xxvii) *y*, *x*−1/2, −*z*+1/2; (xxviii) *y*, −*x*+1/2, *z*−1/2.
